# Could Dry Weight Be Estimated Using Changes in Blood Viscosity in Children on Chronic Hemodialysis?

**DOI:** 10.7759/cureus.48711

**Published:** 2023-11-12

**Authors:** Emre Leventoğlu, Zeynep Ural, Sevcan A Bakkaloğlu

**Affiliations:** 1 Pediatric Nephrology, Gazi University, Ankara, TUR; 2 Nephrology, Kırıkkale Yüksek İhtisas Hospital, Kırıkkale, TUR

**Keywords:** hemorheology, hemodialysis, dry weight, children, blood viscosity

## Abstract

Kidney failure patients on chronic hemodialysis are at risk for cardiovascular complications. Dry weight (DW), one of the dialysis parameters, should be optimized to reduce the complications caused by high blood pressure. By definition, DW is the lowest weight at which patients are clinically euvolemic, not hypotensive or hypertensive after dialysis, and do not require antihypertensives. In hemodialysis patients, DW is achieved by removing fluid from the body through ultrafiltration. Although it is usually determined by trial and error in clinical practice, more objective data are needed. Echocardiography to determine the inferior vena cava diameter and collapse index, bioimpedance analysis to quantify the fluid compartments of the body and blood volume monitoring are among the options. In addition, blood viscosity measurement may be a new method to determine DW.

## Editorial

Kidney failure is a global public health problem causing significant morbidity and mortality. Kidney failure patients on chronic hemodialysis have a poor prognosis due to cardiovascular complications, especially those caused by abnormalities in blood pressure. Therefore, dialysis parameters, especially dry weight (DW), need to be optimized to reduce cardiovascular complications [1].

DW is generally defined as the lowest weight that a patient on chronic hemodialysis can tolerate. Overestimation of DW leads to hypertension, left ventricular hypertrophy and cardiac dysfunction, while underestimation of DW leads to hypotension, asthenia and ischemia. In children, DW may be underestimated due to ponderal growth of patients or overestimated in patients who are malnourished and have weight loss. Therefore, a child’s DW is usually more difficult to assess than that of an adult and usually requires an objective assessment. It is estimated clinically by physical examination, looking for edema or cutaneous folds and blood pressure measurements. In addition, measurement of the cardio-thoracic ratio on chest X-rays, ultrasonic measurement of the diameter of the inferior vena cava, brain natriuretic peptide (BNP)/N-terminal pro-BNP serum levels, multi-frequency bio-impedancemetry, thoracic ultrasound and blood volume monitoring during hemodialysis are helpful methods to determine DW. However, the interindividual variability of many of these methods remains a problem in determining the correct DW. To date, no perfect tool has emerged to precisely assess DW in kidney failure patients on chronic hemodialysis [1].

Viscosity is the resistance of a liquid to flow due to the internal friction between its molecules. Blood is a complex fluid composed of plasma and cells, and blood viscosity is affected by the properties of both these components. The possible role of increased plasma viscosity and decreased cellular deformability in the pathogenesis of atherosclerotic coronary artery disease, a common complication in kidney failure, has been demonstrated [1]. Therefore, measurement of blood viscosity in patients with chronic kidney disease has become even more important. Blood viscosity measurement may be a new method to determine DW. Blood viscosity can be measured as a single parameter by a viscometer, or separately with the help of four parameters such as hematocrit, erythrocyte aggregation, erythrocyte deformability and plasma viscosity [2].

The binding of erythrocytes to each other on their large surfaces by means of high molecular weight macromolecules is called aggregation. Aggregation, which is a physiologic state, is achieved with very weak forces, so it is possible to be reversed. In the case of high hematocrit, there is an increase in aggregation as the interactions of erythrocytes with each other will increase, resulting in an increase in blood viscosity. Conversely, if the hematocrit level is low due to anemia and/or hemodilution, there will be a decrease in aggregation and therefore a decrease in viscosity. Erythrocytes are biconcave disk-shaped. Thanks to their deformability, they can easily pass through capillaries with smaller diameters than themselves. The deformability of erythrocytes is influenced by the flexibility of the membrane skeleton, intracellular viscosity and surface/volume ratio. As water enters the erythrocyte, the volume of the cell increases, and the surface/volume ratio decreases as the surface area remains constant. This is expected to decrease erythrocyte deformability. However, since intracellular viscosity will also decrease with water entry into the cell, some improvement in deformability will occur. The net effect depends on whether the medium is hypotonic or hypertonic and the amount of fluid entering the cell. Plasma viscosity depends on the properties of water, the main substance of plasma, and the macromolecules dissolved in it. If the amount of water in the plasma increases, as in hypervolemia, plasma viscosity decreases. However, an increase in plasma proteins, salt and glucose will lead to an increase in viscosity [2].

Low hematocrit, total protein and albumin values in patients with chronic kidney disease lead to significantly lower blood viscosity levels [3]. In a study by Canaud et al., blood viscosity increased significantly after hemodiafiltration across the entire shear rate spectrum (from 24.86 to 33.41 when the shear rate was 0.15 s; from 12.75 to 17.25 when the shear rate was 0.94 s; from 3.73 to 4.65 when the shear rate was 128 s) [4]. In another study by Dhar et al., the mean blood viscosity was found to be significantly higher after dialysis than pre-dialysis. In that study, high blood viscosity above acceptable limits had been correlated with increased incidence of access failure and vascular disease. Recurrent increases in blood viscosity with each dialysis treatment have been reported to contribute to vascular dysfunction in that population [5].

Based on these studies, it is thought that the lower and upper limits of blood viscosity can be determined with only a few milliliters of blood samples before and after dialysis in pediatric chronic hemodialysis patients, where there are many confounding factors for the determination of DW, and thus, blood viscosity can be used as an important tool in estimating the DW of pediatric patients.

## References

[1] Hothi DK, Harvey E, Goia CM, Geary DF (2008). Evaluating methods for improving ultrafiltration in pediatric hemodialysis. Pediatr Nephrol.

[2] Dikmenoğlu N, Ciftçi B, Ileri E, Güven SF, Seringeç N, Aksoy Y, Ercil D (2006). Erythrocyte deformability, plasma viscosity and oxidative status in patients with severe obstructive sleep apnea syndrome. Sleep Med.

[3] Buyan N, Akçaboy M, Göktaş T (2017). Effects of whole blood viscosity and plasma NOx on cardiac function and cerebral blood flow in children with chronic kidney disease. Turk J Med Sci.

[4] Canaud B, Rodriguez A, Chenine L (2010). Whole-blood viscosity increases significantly in small arteries and capillaries in hemodiafiltration. Does acute hemorheological change trigger cardiovascular risk events in hemodialysis patient?. Hemodial Int.

[5] Dhar P, Eadon M, Hallak P, Munoz RA, Hammes M (2012). Whole blood viscosity: effect of hemodialysis treatment and implications for access patency and vascular disease. Clin Hemorheol Microcirc.

